# Supplementary material for: Auditory working memory mechanisms mediating the relationship between musicianship and auditory stream segregation
Source: Front Psychol. 2025 Mar 28;16:1538511. doi: 10.3389/fpsyg.2025.1538511 (PMC11989347; doi:10.3389/fpsyg.2025.1538511)
Supplement: Supplementary file 1 [file Supplementary_file_1.docx]

**Supplementary Material**


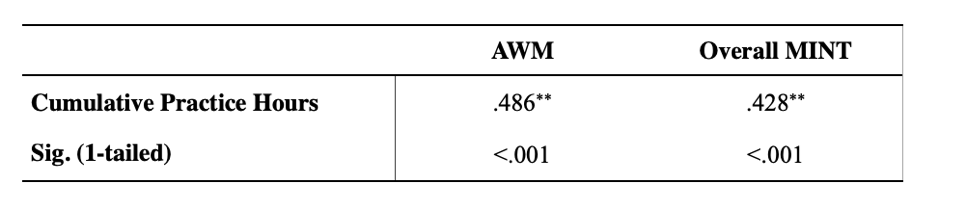


Table S1*.* Experiment 1. Spearman’s rho for cumulative practice hours vs. AWM task performance and cumulative practice hours vs. overall MINT performance.


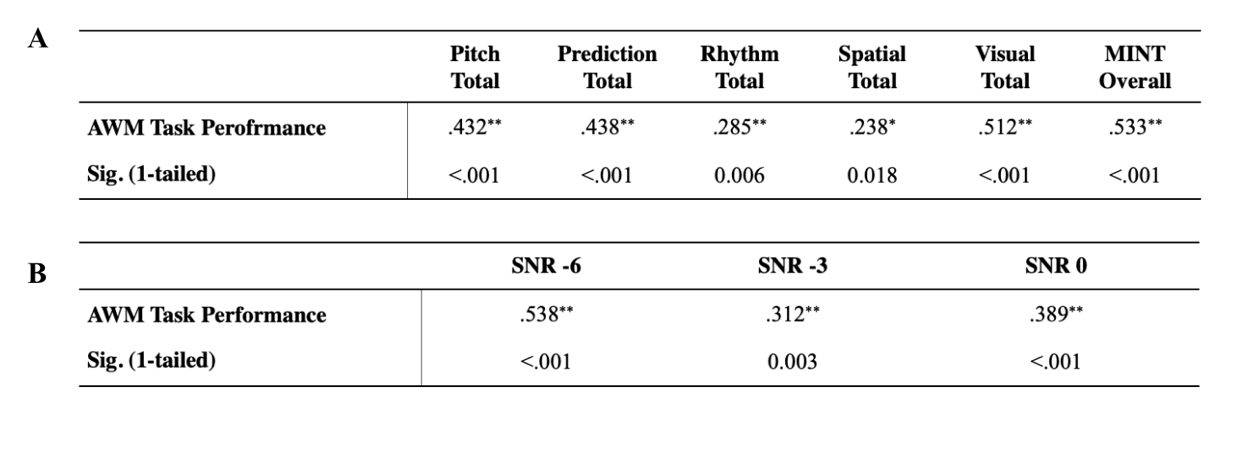


Table S2*.* Experiment 1. (A) AWM Task Performance vs. MINT Conditions. Spearman’s rho based on AWM task percent correct and average MINT scores for the corresponding condition.

(B) AWM Task Performance vs. MINT SNR Levels. Spearman’s rho based on AWM task percent correct and average MINT scores for each SNR level across conditions.


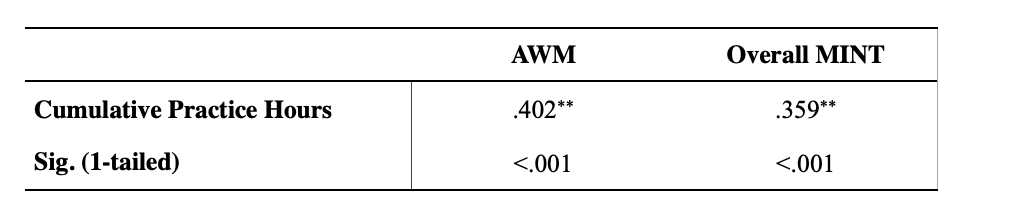


Table S3*.* Experiment 2. Spearman’s rho for cumulative practice hours vs. AWM task performance and cumulative practice hours vs. overall MINT performance.


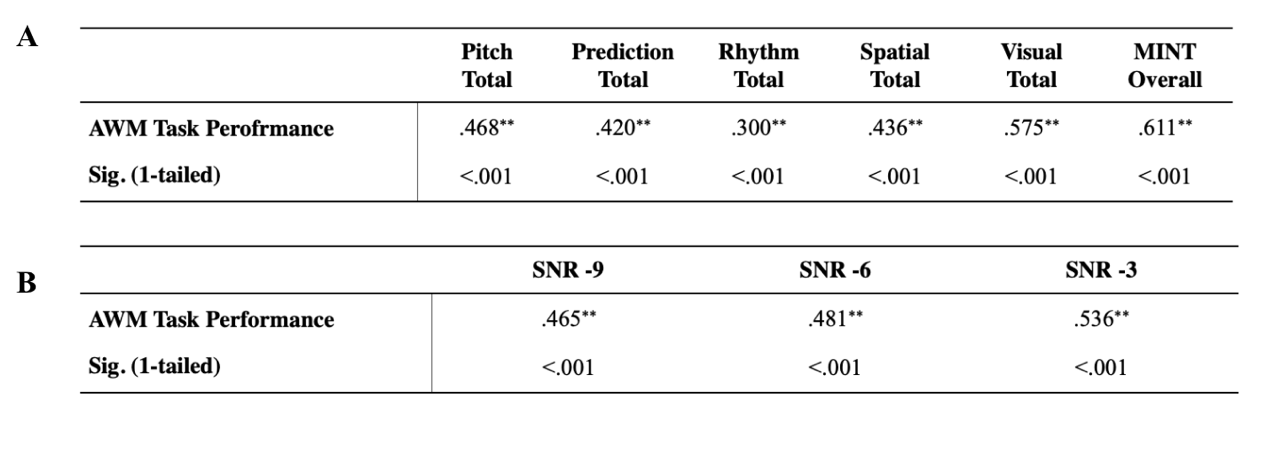


Table S4*.* Experiment 2. (A) AWM Task Performance vs. MINT Conditions. Spearman’s rho based on AWM task percent correct and average MINT scores for the corresponding condition.

(B) AWM Task Performance vs. MINT SNR Levels. Spearman’s rho based on AWM task percent correct and average MINT scores for each SNR level across conditions.
